# Notes From the Field: Use of Emergency Medical Service Data to Augment COVID-19 Public Health Surveillance in Montgomery County, Maryland, From March to June 2020

**DOI:** 10.2196/22331

**Published:** 2020-07-31

**Authors:** David R Sayers, Scott T Hulse, Bryant J Webber, Timothy A Burns, Anne L Denicoff

**Affiliations:** 1 Uniformed Services University of the Health Sciences Bethesda, MD United States; 2 Montgomery County Fire and Rescue Service Gaithersburg, MD United States; 3 Montgomery County Public Health Emergency Preparedness and Response Program Rockville, MD United States

**Keywords:** SARS-CoV-2, COVID-19, public health, surveillance, prediction, emergency medical service, EMS, pulse oximetry, testing

## Abstract

Epidemiologic and syndromic surveillance metrics traditionally used by public health departments can be enhanced to better predict hospitalization for coronavirus disease (COVID-19). In Montgomery County, Maryland, measurements of oxygen saturation (SpO_2_) by pulse oximetry obtained by the emergency medical service (EMS) were added to these traditional metrics to enhance the public health picture for decision makers. During a 78-day period, the rolling 7-day average of the percentage of EMS patients with SpO_2_ <94% had a stronger correlation with next-day hospital bed occupancy (Spearman ρ=0.58, 95% CI 0.40-0.71) than either the rolling 7-day average of the percentage of positive tests (ρ=0.55, 95% CI: 0.37-0.69) or the rolling 7-day average of the percentage of emergency department visits for COVID-19–like illness (ρ=0.49, 95% CI: 0.30-0.64). Health departments should consider adding EMS data to augment COVID-19 surveillance and thus improve resource allocation.

## Introduction

On March 5, 2020, Montgomery County, Maryland, a densely populated county neighboring Washington, DC, reported its first cases of coronavirus disease (COVID-19); this prompted the county health department to develop a daily surveillance report [1]. By March 27, this report included the following information: daily and cumulative confirmed COVID-19 cases; percentage of reverse transcription polymerase chain reaction (RT-PCR) tests positive for severe acute respiratory syndrome coronavirus 2 (SARS-CoV-2), the virus that causes COVID-19; acute and intensive care unit beds occupied in the county’s seven hospitals; daily emergency department encounters for COVID-19–like illness; and daily emergency medical service (EMS) calls and acuity indicators, including the number of patients with a pre-hospital pulse oximetry value (SpO_2_) below 94% [2]. Epidemiologic data were retrieved from the Maryland Department of Health. Emergency department syndromic data were retrieved from the Montgomery County Electronic Surveillance System for the Early Notification of Community-based Epidemics (ESSENCE) using the COVID-19–like illness query published by the National Syndrome Surveillance Program; this query is defined as fever plus cough, difficulty breathing, or shortness of breath, and it includes International Statistical Classification of Diseases, Tenth Revision (ICD-10) codes for COVID-19 [3]. EMS data were provided by the Montgomery County Fire and Rescue Service.

**Figure 1:**
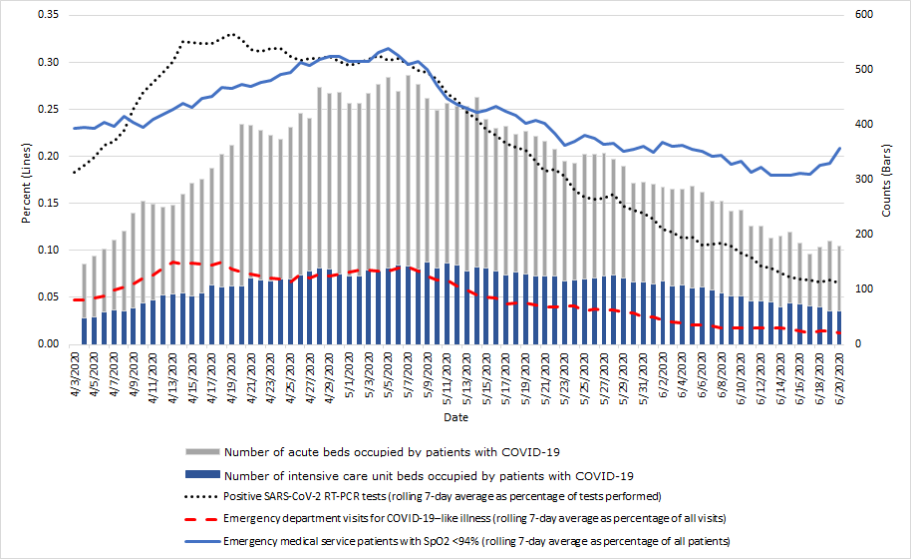
Metrics associated with hospital bed occupancy for COVID-19 in Montgomery County, Maryland, from April 3 to June 20, 2020.

As the situation unfolded, it was noted that the percentage of EMS patients with SpO_2_ <94% tracked closely with the number of hospital beds occupied by patients with COVID-19 in the county (Figure 1). It was postulated that this metric, in addition to typical epidemiologic and syndromic surveillance data, may be beneficial for hospital utilization forecasting.

## Methods

The relationship between prehospital hypoxemia and next-day hospital bed occupancy for COVID-19 was assessed using the Spearman rank-order correlation. Prehospital hypoxemia was defined as the rolling 7-day average of the percentage of EMS patients with SpO_2_ <94%. The Spearman rank-order correlation was also used to assess the correlation between the rolling 7-day average of the percentage of RT-PCR tests that were positive for SARS-CoV-2 and next-day hospital bed occupancy, as well as the correlation between the rolling 7-day average of the percentage of emergency department visits for COVID-19–like illness and next-day hospital bed occupancy. Correlations were computed using SAS 9.4 (SAS Institute) with 95% CIs based on the Fisher z transformation. This study was approved as exempt research by the Maryland Department of Health Institutional Review Board (protocol #20-32).

## Results

During the 78-day period from April 3 to June 19, 2020, the correlation coefficient (ρ) between the rolling 7-day average of the percentage of EMS patients with SpO_2_ <94% and the total hospital bed occupancy on the following day (ie, from April 4 to June 20) was 0.58 (95% CI 0.40-0.71). This correlation was stronger than those for the two other metrics commonly used to assess COVID-19 trajectory in a community: the rolling 7-day average of the percentage of positive tests (ρ=0.55, 95% CI 0.37-0.69) and the rolling 7-day average of the percentage of emergency department visits for COVID-19–like illness (ρ=0.49, 95% CI 0.30-0.64).

## Discussion

To reduce morbidity and mortality associated with the ongoing pandemic, government authorities and health care administrators must anticipate demands for hospital beds, equipment, and treatments [4]. These leaders will continue to rely on public health metrics to anticipate surges in the number of patients with COVID-19. The value of these metrics increases with their predictive ability and their nearness to real time [3,5]; ideally, extant metrics can be adopted without implementing novel data collection infrastructure [6].

Prehospital pulse oximetry may meet these requirements and surpass traditional surveillance measures for predicting COVID-19 hospitalizations for at least four reasons. First, by requiring two sets of vital signs and by documenting SpO_2_ for nearly every patient encounter regardless of presentation or working diagnosis, EMS has established a metric that is comprehensively ascertained and internally valid. Second, these data are usually generated before those from traditional health care sources, such as emergency department assessments and RT-PCR test results. Third, because hypoxemic patients are more likely than asymptomatic or mildly symptomatic patients to be hospitalized, the predictive criterion validity of SpO_2_ may surpass that of RT-PCR test positivity [7]. Fourth, although syndromic surveillance provides some information on disease severity, patient acuity indicators are not consistently populated in the Montgomery County syndromic system, and emergency department pulse oximetry measurements may be affected by oxygen administration in the prehospital environment.

In Montgomery County, Maryland, the 7-day rolling average of the percentage of EMS patients with SpO_2_ <94% correlated well with next-day hospitalizations for COVID-19. State and county health departments should consider tracking the hypoxemia status of prehospital patients by using EMS data to augment surveillance and improve their COVID-19 response.

## Abbreviations

COVID-19: coronavirus disease
EMS: emergency medical service
ESSENCE: Electronic Surveillance System for the Early Notification of Community-based Epidemics
ICD-10: International Statistical Classification of Diseases, Tenth Revision
RT-PCR: reverse transcription–polymerase chain reaction
SARS-CoV-2: severe acute respiratory syndrome coronavirus 2
